# Curculigoside Protects against Excess-Iron-Induced Bone Loss by Attenuating Akt-FoxO1-Dependent Oxidative Damage to Mice and Osteoblastic MC3T3-E1 Cells

**DOI:** 10.1155/2019/9281481

**Published:** 2019-12-17

**Authors:** Quanlong Zhang, Lu Zhao, Yi Shen, Yuqiong He, Gang Cheng, Ming Yin, Qiaoyan Zhang, Luping Qin

**Affiliations:** ^1^College of Pharmaceutical Sciences, Zhejiang Chinese Medical University, Hangzhou 310050, China; ^2^School of Pharmacy, Xuzhou Medical University, Xuzhou 221004, China; ^3^School of Pharmacy, Fujian Chinese Medical University, Fujian 350122, China; ^4^School of Pharmacy, Second Military Medical University, Shanghai 200433, China; ^5^School of Pharmacy, Shanghai Jiao Tong University, Shanghai 200052, China

## Abstract

**Summary:**

The present investigation found that curculigoside (CUR) can prevent excess-iron-induced bone loss in mice and cells through antioxidation and inhibiting excess-iron-induced phosphorylation of the Akt-FoxO1 pathway. CUR can attenuate the decreasing of cell viability, enhance autophagy, potentiate the antioxidant effect, and reduce apoptosis in MC3T3-E1 cells treated with excess iron through regulating the expression of FoxO1 target gene.

**Introduction:**

Oxidative stress induced by iron overload is an important factor involved in primary osteoporosis disease and iron overload-related diseases. Curculigoside (CUR), a phenolic glycoside found abundantly in *Curculigo orchioides* Gaertn., has been demonstrated to possess antioxidant and antiosteoporotic properties. The aim of the present study is to explore the underlying molecular mechanism of CUR on excess-iron-induced bone loss in mice and osteoblastic MC3T3-E1 cells.

**Methods:**

An iron-overload mice model was used to study the protective effects of CUR on bone loss induced by oxidative stress. Serum bone metabolism markers and antioxidant enzymes were also measured. To explore the antioxidant mechanism of CUR, the MC3T3-E1 osteoblastic cell line was used.

**Results:**

In vivo studies showed that BMD and microarchitectural parameters were improved after a 3-month administration of CUR. CUR improved the biochemical parameters related to bone metabolism and the expressions of Runx2, OCN, and type 1 collagen and increased the formation of bone-mineralized nodules *in vitro*. CUR also inhibited ROS generation and increased the activities of antioxidant enzymes both *in vivo* and *in vitro* treated with excess iron. CUR can upregulate the level of FoxO1 and Nrf2, downregulate the level of p53 and the phosphorylation level of FoxO1, improve nuclear translocation of FoxO1, probably by inhibiting the IGFR/AKT signaling pathway, then increased cell viability and autophagy, and reduced apoptosis of MC3T3-E1 cells treated with excess iron by regulating the expression of FoxO1 target genes MnSOD, Gadd45a, Bim, FasL, and Rab7.

**Conclusions:**

These results demonstrated that CUR was able to alleviate bone loss induced by oxidative stress resulting from iron overload, suggesting its potential use for the treatment of primary osteoporosis and bone loss in iron-overload-related diseases.

## 1. Introduction

Osteoporosis is a chronic disease caused by many factors, characterized by bone mass decline and trabecular architecture deterioration leading to an increased risk of fracture. Both estrogen deficiency and aging can induce oxidative stress, thus increasing the generation of reactive oxygen species (ROS), decreasing osteoblastic bone formation, and elevating the level of osteoclastic bone resorption [1, 2]. It is therefore generally believed that aging and associated increase in ROS are the primary contributing factors of osteoporosis, and some antioxidants such as vitamin E, vitamin C, and N-acetylcysteine (NAC) have been canonized as alternative therapeutic agents for osteoporosis.

Curculigoside (CUR) is a naturally occurring phenolic glycoside compound from *Curculigo orchioides* Gaertn. that has long been used for the treatment of osteoporosis and postmenopausal syndrome in China. Several biologic effects of CUR have been reported, including antioxidant, antiaging, immunomodulatory, anticancer, and bone-protective activities [3–6]. The bone-protective effects of CUR have been evidenced in *vivo* and *in vitro* studies [7]. CUR was reported to reduce loss of bone mass in ovarietomized rats by upregulating ER-dependent bone morphogenetic protein 2 (BMP-2), enhancing osteogenesis and inhibiting adipogenesis in murine pluripotent mesenchymal cells or bone marrow-derived mesenchymal stem cells (MSCs) [8–10]. Our previous study found that CUR could increase osteoblastic proliferation, differentiation, and calcification of mouse osteoblastic MC3T3-E1 cells [9], and attenuate dysfunction and oxidative damage induced by hydrogen peroxide in calvarial osteoblasts by decreasing ROS production and lipid peroxidation and increasing the activities of antioxidant enzymes such as superoxide dismutase (SOD) and glutathione peroxidase (GSH-PX) in H_2_O_2_-induced osteoblast impairment [10]. Interestingly, oral administration of CUR also significantly enhanced learning performance and ameliorated bone loss in APP/PS1-mutated transgenic mice through decreasing the ROS level and increasing the capacity of antioxidative enzymes [11]. These findings demonstrated that the bone-protective effect of CUR may be associated with its antioxidative activity. However, the underlying mechanism remains obscure.

Oxidative stress is known to be involved in the pathogenesis of osteoporosis by affecting the generation and survival of osteoblasts (OB) and osteoclasts (OC). The transcriptional factor and antioxidant enzymes protect cells and the body against oxidative insults. FoxOs, also known as Fork head box, including FoxO1, FoxO3, FoxO4, and FoxO6, are antioxidant transcriptional factors. FoxO1, which is reported to regulate oxidative stress, osteoblast proliferation, and bone formation, may be a key modulator in redox signaling and bone homeostasis [12, 13]. FoxO1 is activated by ROS, translocates into the nucleus, where it upregulates the activities of antioxidant enzymes GSH-PX and SOD, and mediates cell antioxidant stress, apoptosis, and autophagy by controlling the expression of other genes, such as Gadd45, Rab7, Bim, and FasL [14, 15]. Hence, FoxO1 represents a major cell defense mechanism against oxidative insults in osteoblasts.

Oxidative stress results from multiple factors including aging, estrogen deficiency, and accumulation of some detrimental substances such as some metals. Iron is one of the most abundant transition metals in the human body and plays a key role in various biological processes. However, excessive iron is harmful to organ functions or even causes potentially irreversible organ damage. Iron-induced damage to organs is mainly due to the function of iron in catalyzing ROS formation via Fenton reaction. Some recent studies [16] demonstrated that elevation of iron level is a risk factor for osteoporosis, and reducing iron overload has been demonstrated to benefit bone cell metabolism and improve the bone mass. In addition, many experiments showed that the iron-overload could alter the bone microarchitectures including the trabecular number, thickness, and bone volume fraction, and increase bone resorption in mice [17]. Therefore, the mouse or rat excess-iron treatment models could be used to investigate the effects of some drugs on bone loss and osteoporosis, especially those with antioxidative capacities [18].

Knowing that FoxO1 is a positive regulator of bone formation through its resistance to oxidative stress in osteoblast [12], and iron overload induces oxidative stress, bone loss, and osteoporosis, we postulated that CUR may be able to exert an effect on FoxO1 and affect the transcription of target genes to regulate oxidative stress and bone remodeling. The aim of the present study was to investigate the bone-protective effect of CUR in iron-overload mice and osteoblastic damaged by excess iron, and explore the underlying mechanism via the AKT-FoxO1 pathway. The results of the present study showed that CUR could reverse the bone loss and oxidant damage under the iron-overload condition through the IGFR/AKT/FoxO1 pathways both *in vivo* and *in vitro*. Our data may provide new insights into the molecular mechanism underlying the important antiosteoporotic effect of CUR.

## 2. Materials and Methods

### 2.1. Reagents

CUR was prepared in our laboratory and identified with UV, IR, NMR, and MS by comparing the obtained data with the published literature. Iron dextran, N-acetyl-L-cysteine (NAC), desferrioxamine (DFO), ferric ammonium citrate (FAC), and all other chemicals were purchased from Sigma-Aldrich (St. Louis, MO, USA).

Antiosteocalcin (OCN) antibody was purchased from Millipore (Bedford, MA). The mouse anti-FoxO1 antibody, FoxO1 (C29H4) rabbit mAb, Nrf-2, p62, p53, Beclin-1, and anti-beta-catenin were purchased from Cell Signaling Technology (Beverly, MA). Col I, Runx2, SOD, P66, p-P66, p-JNK, JNK, GLUT1, GLUT3, IGFR, Akt, p-Akt, p-FoxO1, Bcl-2, BAX, caspase-3, LC3B, histone H3, and GAPDH antibodies were purchased from Abcam (Cambridge, MA). Goat anti-mouse IgG (H+L) Cross-Adsorbed Secondary antibody, DyLight 488, Cyanine 3, and DAPI were from Life Technologies. The alkaline phosphatase (ALP), OCN, and tartrate resistant acid phosphatase-5b (TRACP-5b), IL-6, TNF-*α*, superoxide dismutase (SOD), catalase (CAT), and malondialdehyde (MDA) kits were purchased from Jiancheng Bioengineering Institute (Nanjing, China). The ROS kit and Bradford protein measurement kit were purchased from Solarbio (Beijing, China). Dimethyl sulfoxide and fetal bovine serum were purchased from Sigma (Sigma-Aldrich, USA). *α*-MEM, Dulbecco's modified Eagle's medium (DMEM), and penicillin/streptomycin were purchased from Hyclone (Thermo, USA).

### 2.2. Animal Experiments

Maintenance, use, and treatment of all animals in this study were in accordance with the accepted standards of the Ethics Committee at Shanghai Jiao Tong University (Approval No. SYXK 2012-0017, Shanghai, China). Sixty male C57/BL6 mice aged 2 months and weighing 25-30 g were obtained from Shanghai Slack Animal Co., Ltd. (Shanghai, China). After a 2-day acclimatization, the 60 animals were equally randomized into six groups: a normal control group, a model control group (100 mg/kg iron dextran, i.p. per week), a positive control group (100 mg/kg iron dextran, i.p. per week +100 mg/kg NAC, i.g. per day), a low-dose CUR group (100 mg/kg iron dextran, i.p. per week +25 mg/kg CUR, i.g. per day), a middle-dose CUR group (100 mg/kg iron dextran, i.p. per week +50 mg/kg CUR, i.g. per day), and a high-dose CUR group (100 mg/kg iron dextran, i.p. per week +CUR 100 mg/kg, i.g. per day). Except for those in the normal control group, all the other animals were treated intraperitoneally (i.p.) with iron dextran (100 mg/kg) once a week for 3 months. The mice in the normal and model control groups were orally administered with 0.5% CMC-Na, and others were orally given NAC or CUR according to the experimental protocols. After a 90-day treatment, all mice were euthanized with excessive amounts of anesthetics. Blood samples were collected from the carotid artery and centrifuged at 1400×g for 10 min to collect the sera, which were then stored at −20°C prior to biomarker assay.

The left femurs were collected for micro-CT scanning as described previously [19]. The bone mineral content (BMC), bone mineral density (BMD), structure model index (SMI), bone volume fraction (BVF, ratio of bone volume to tissue volume, BV/TV), bone surface to bone volume (BS/BV), trabecular thickness (Tb.Th), trabecular separation (Tb.Sp), trabecular number (Tb.N), and connectivity density (Conn. D) were determined. The Explore Reconstruction Utility (GE Healthcare, USA) was used for 3D reconstruction and data processing.

Three-point bending testing was performed using a Dynamic Mechanical Analyzer (Shimadzu, Japan) to determine the mechanical properties of the right femurs of the mice, including elastic load, maximum load, elastic stress, maximum stress, and modulus of elasticity.

Liver homogenate was centrifuged at 3000 g/min for 10 min. The supernatant was collected for detecting SOD (supernatant dismutase), CAT (catalase), GSH-Px (glutathione peroxidase), and MDA (malondialdehyde) activities by relative commercial kits (Jiancheng, Nanjing, China).

Serum samples were centrifuged at 3000 g/min for 10 min. The supernatant was collected for ELISA detection. Contents of TNF-*α*, IL-6, TRACP 5b, and OCN levels were detected according to the instructions of ELISA kit (Abcam, USA).

### 2.3. Cell Culture and Experimental Protocols in Osteoblasts

MC3T3-E1 cells derived from newborn mouse calvaria were purchased from the typical Culture Committee Cell Library of the Chinese Academy of Sciences (Shanghai, China) and maintained in *α*-MEM containing 10% FBS in a humidified atmosphere of 5% CO_2_ at 37°C. Cells were harvested when they reached approximately 80% confluence to conduct the following experiments. MC3T3-E1 cells were treated with CUR (10 *μ*M), NAC (1000 *μ*M), and iron chelator desferrioxamine (DFO, 100 *μ*M) for 2 h, and then treated with ferric ammonium citrate (FAC, 500 *μ*M) to induce oxidative stress. All the experiments were repeated 3 times.

### 2.4. Proliferation Assay by MTT Method

To examine the effect of CUR on FAC-treated MC3T3-E1 cell proliferation, cells were seeded in 96-well plates at 5 × 10^3^ cells/well, cultured overnight, and then treated with CUR (10 *μ*M), NAC (1000 *μ*M), or DFO (100 *μ*M) with or without FAC (500 *μ*M) for 48 h. 20 *μ*l MTT (5 mg/ml) was added in phosphate-buffered solution (pH 7.4) at the end of the treatment. After additional 4 h incubation, the supernatant was abandoned. 150 *μ*l dimethyl sulfoxide (DMSO) was added to each well to dissolve the formazan crystals that formed in the cells. The absorbance was measured at 570 nm.

### 2.5. Observation and Analysis of Bone Mineralization

MC3T3-E1 cells were seeded in 24-well plates at 1 × 10^5^ cells, allowed to confluence overnight, and then cultured in osteoblast differentiation medium containing 10 nM dexamethasone, 50 *μ*g/ml ascorbic acid, and 10 mM *β*-glycerophosphate for 15 days, with the medium replaced at a 3-day interval. Then, cells were incubated in osteoblast differentiation medium containing CUR (10 *μ*M), NAC (1000 *μ*M), or DFO (100 *μ*M) with or without FAC (500 *μ*M) for additional 6 days, washed with PBS, fixed with 4% paraformaldehyde for 30 min, and stained with 0.1% AR-S-Tris-HCl (pH 8.3) for 30 min at 37°C to observe bone nodules under the microscope. The images were photographed.

### 2.6. Determination of SOD, CAT, MDA, and GPx in MC3T3-E1 Cells

MC3T3-E1 cells were plated in 24-well plates at a density of 5 × 10^4^ cells, allowed to confluence overnight, and then treated with CUR (10 *μ*M), NAC (1000 *μ*M), or DFO (100 *μ*M) with or without FAC (500 *μ*M) for 48 h. The cells were collected using a rubber scraper, and proteins were extracted with RIPA buffer. The concentration of proteins from MC3T3-E1 cells was determined using a BCA protein assay kit (Jiancheng, Nanjing, China). The activities of CAT, SOD, and GPx, and the concentration of MDA in cell lysates were examined according to the manufacturer's recommendations (Beyotime Biotechnology, Shanghai, China).

### 2.7. Measurement of Intracellular ROS Levels

MC3T3-E1 cells were seeded in 24-well plates at a density of 5 × 10^4^ cells, allowed to confluence overnight, treated with CUR (10 *μ*M), NAC (1000 *μ*M), or DFO (100 *μ*M) with or without FAC (500 *μ*M) for 48 h, and incubated with 5 *μ*M 2′,7′-dichlorodihydrofluoresceindiacetate (H2DCFDA), a ROS indicator, for 30 min at room temperature. Fluorescence was monitored under an inverted fluorescent microscope (DMI3000 B; Leica) using excitation/emission wavelength of 535 nm. Signal increases are presented as the peak/basal fluorescence ratio (F/F0).

### 2.8. RT-Quantitative Real-Time PCR (RT-qPCR)

MC3T3-E1 cells were plated in 6-well culture plates at a density of 1 × 10^5^ cells and allowed to attach overnight, and then treated with CUR (10 *μ*M), NAC (1000 *μ*M), or DFO (100 *μ*M) with or without FAC (500 *μ*M) for 48 h. Total RNA was extracted using the RNA extraction kit (Takala, Japan). The concentration and quality of RNA were measured using a NanoDropND-1000 system (Thermo Fisher Scientific Inc.). For RT-qPCR expression analysis, 500 ng RNA was reversed using PrimeScript RT Reagent Kit with cDNA Eraser Kit (Takala, Japan) according to the manufacturer's instructions. The primers used for SYBR Green RT-qPCR are shown in Supplementary [Supplementary-material supplementary-material-1]. The quantitative expression analysis was performed using an ABI 7500 system (Life Tech, USA) and predesigned SYBR green expression assays (Bio-Rad, USA). GAPDH expression was used as an internal control. Relative quantification was performed according to the *^ΔΔ^*CT method.

### 2.9. Western Blotting and ELISA Analysis

MC3T3-E1 cells were seeded in 6-well plates after overnight incubation at 37°C at a density of 1 × 10^5^ cells per well. Then, cells were cultured with DFO (100 *μ*M), NAC (1000 *μ*M), or CUR (10 *μ*M) for 2 h, and subjected to FAC (500 *μ*M) for 72 h. Cells were collected using a rubber scraper, and proteins were extracted with RIPA buffer. The nucleoprotein and cytoplasmic protein from MC3T3-E1cells were separated using an extraction kit (Jiancheng, Nanjing, China) and their concentrations were determined using a BCA protein assay kit (Jiancheng, Nanjing, China). Protein (20–30 *μ*g) was loaded onto a 12% gel using 25 *μ*g proteins per lane and transferred to PVDF membranes. The membranes were blocked, incubated with primary antibodies (Col I, Runx2, OCN, OPG, RANKL, SOD, catalase, p-P66, p-JNK, JNK, GLUT1, GLUT3, IGFR, Akt, p-Akt, FoxO1, p-FoxO1, Bcl-2, BAX, caspase-3, LC3B, Nrf-2, p53, p62, Beclin-1, histone H3, and GAPDH) overnight at 4°C, washed with PBST (0.1% Tween-20), and incubated for 1 h with the corresponding secondary antibody conjugated with horseradish peroxidase. The membranes were washed three times with PBST and visualized using an enhanced ECL chemoluminescent Western blotting detection system (Millipore, USA). The band density was quantified using ImageJ soft (LI-COR Biosciences).

### 2.10. Apoptosis and Mitochondrial Membrane Potential Assay

To examine the effect of CUR on the mitochondrial membrane potential in FAC treated MC3T3-E1 cells, cells were seeded in 96-well plates at a density of 5 × 103 cells per well and allowed to attach overnight. Then, cells were treated with CUR (10 *μ*M), NAC (1000 *μ*M), or DFO (100 *μ*M) with or without FAC (500 *μ*M) for 48 h. After addition of 100 *μ*l JC-1 staining solution per well, cells were incubated for 15-30 min. The absorbance was measured at 590 nm.

### 2.11. Statistical Analyses

Data are expressed as means ± standard deviation (SD) of at least three independent experiments. All statistical analyses were performed by one-way analysis of variance and Dunnett's *t*-test using SPSS 13.0 software (SPSS, Chicago, USA). *P* values < 0.05 were considered statistically significant. All graphs were drawn using GraphPad Prism (version 6.0 for Windows).

## 3. Results

### 3.1. CUR Attenuates Excess-Iron-Induced Bone Loss in Mice and Osteoblastic MC3T3-E1 Cells

**Figure 1:**
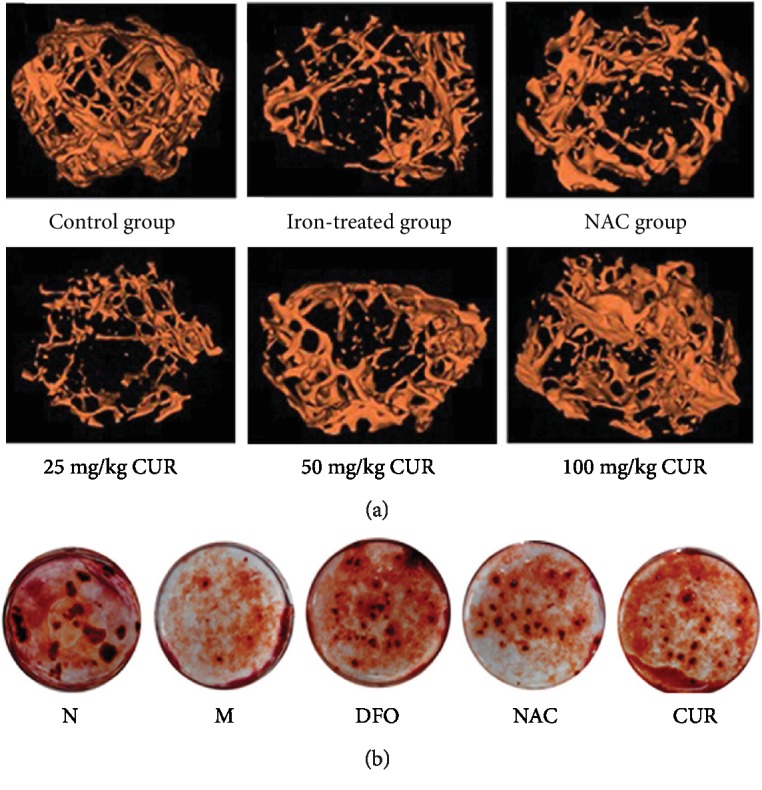
Effects of CUR on bone architecture properties of the femurs in iron-overload mice and formation of bone-mineralized nodules in osteoblastic MC3T3-E1 cells treated with excess iron. (a) Representative 3D reconstructed images analyzed by micro-CT. C57/BL6 male mice aged 2 months were treated with i.p. iron dextran (100 mg/kg) once a week for 3 months and simultaneously given NAC and various doses of CUR, and subsequently, bone architecture properties of the femur were analyzed by micro-CT. (b) Bone-mineralized nodules in osteoblastic MC3T3-E1 cells. Osteoblastic MC3T3-E1 cells were incubated in osteoblast differentiation medium containing CUR (10 *μ*M), NAC (1000 *μ*M), or DFO (100 *μ*M) with or without FAC for additional 6 days and then washed with PBS, fixed with 4% paraformaldehyde for 30 min, and stained with 0.1% AR-S-Tris-HCl (pH 8.3) for 30 min at 37°C. Bone nodules were observed and photographed with a microscope. N: normal control group, cells were treated with DMSO; M: model group, cells were treated with FAC (500 *μ*M); DFO: cells were treated with DFO (100 *μ*M) and FAC (500 *μ*M); NAC: cells were treated with NAC (1000 *μ*M) and FAC (500 *μ*M); CUR: cells were treated with CUR (10 *μ*M) and FAC (500 *μ*M).

Alteration in the bone mass architecture and mechanical properties is often used to predict the risk of bone fracture. The micro-CT analysis of distal femoral diaphysis indicated that the femoral BMD was decreased in mice after administration of iron dextran for 90 days as compared with that in the normal control group (Table 1, Figure 1(a)). As a positive control, NAC (500 mg/kg) led to an obvious increase in BMD. BMD in all mice administered with various amounts of CUR, especially those in the high-dose group (100 mg/kg), was significantly increased as compared with that in iron-overload mice. The results of bone tissue morphological parameters showed that excess iron resulted in a significant decrease in BVF, trabecular number, and thickness, with a concomitant increase in bone surface to bone volume (BS/BV) and trabecular spacing, while CUR (100 mg/kg) and NAC (500 mg/kg) treatment alleviated the morphological alteration of bone induced by iron excess in mice. In addition, the mice in the iron-overload group showed thinner cortices and decreased cortical areas compared with the normal control, while CUR and NAC revealed a remarkable effect on cortical bone in the femur of iron-overload mice.

The analytical results of three-point bending test also exhibited that 90-day treatment with iron overload significantly reduced elastic load, maximum load, elastic stress, maximum stress, and elastic modulus of the mouse femur (Table 1), while CUR (100 mg/kg) and NAC (500 mg/kg) improved all parameters related to bone strength and inhibited the deterioration of bone mechanical properties compared with the iron-overload control, indicating that CUR significantly improved the bone mechanical strength in the iron-overload osteoporosis model.

Treatment of osteoblastic MC3T3-E1 cells with FAC significantly decreased bone mineral nodule formation. Pretreatment with CUR (10 *μ*M), antioxidant NAC (1000 *μ*M), or iron chelator desferrioxamine (DFO, 100 *μ*M) for 24 h reversed the reduction in the mineralization of osteoblastic MC3T3-E1 cells treated with FAC (Figure 1(b)).

### 3.2. CUR Regulates Bone Metabolism in Iron-Overload Mice and Osteoblastic MC3T3-E1 Cells Damaged with Excess Iron

**Figure 2:**
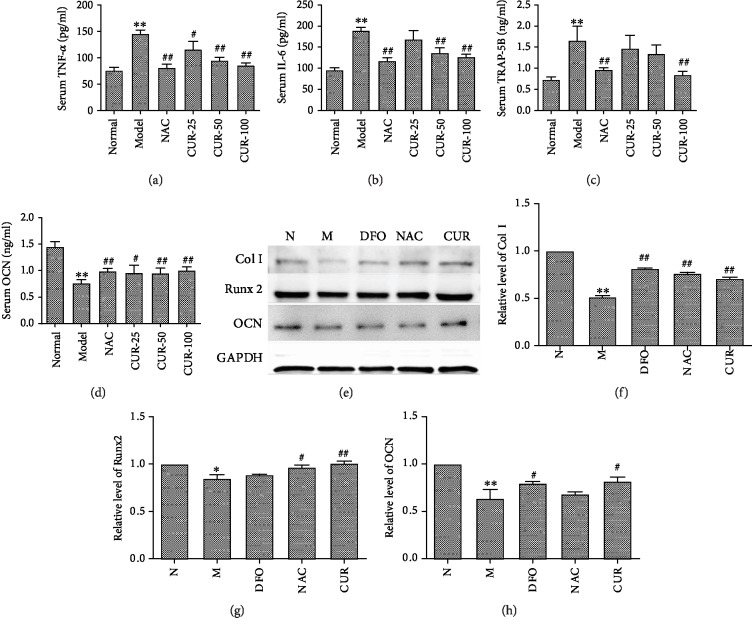
The regulatory effect of CUR on bone metabolism in iron-overload mice and MC3T3-E1 cells treated with excess iron. (a–d) Serum level of TNF-*α*, IL-6, TRACP-5b, and OCN in iron-overload mice assayed with an ELISA kit. (e–h) The expression of Col1, Runx2, and OCN in MC3T3-E1 cells treated with excess iron. Results are represented as the mean ± SD. (*n* = 10 in animal experiments; *n* = 6 in osteoblast experiments), ^∗^*P* < 0.05 and ^∗∗^*P* < 0.01, compared with the normal control group; ^#^*P* < 0.05 and ^##^*P* < 0.01, compared with the model group. N: normal control group, cells were treated with DMSO; M: model group, cells were treated with FAC (500 *μ*M); DFO: cells were treated with DFO (100 *μ*M) and FAC (500 *μ*M); NAC: cells were treated with NAC (1000 *μ*M) and FAC (500 *μ*M); CUR: cells were treated with CUR (10 *μ*M) and FAC (500 *μ*M).

In this study, excess iron induced oxidative stress and caused the imbalance of bone metabolism. As shown in Figures 2(a)–2(d), iron overload increased the level of TRACP-5b, IL-6, and TNF-*α*, and decreased the serum level of OCN, indicating that iron overload significantly enhanced bone resorption and inhibited bone formation in mice. CUR and antioxidant NAC treatment decreased the level of IL-6, TNF-*α*, and TRACP-5b, and enhanced the level of OCN in serum of iron-overload mice. These findings suggest that CUR was involved in the regulation of bone metabolism disturbance induced by iron overload.

Additionally, treatment of osteoblastic MC3T3-E1 cells with FAC significantly decreased the expression of Runx 2, osteocalcin (OCN), and collagen I (Figures 2(e)–2(h)), indicating that excess iron suppressed osteoblastic differentiation. Pretreatment with CUR (10 *μ*M), antioxidant NAC (1000 *μ*M), or DFO (100 *μ*M) for 72 h significantly protected osteoblasts against the reduction in Runx2, collagen I, and OCN induced by FAC (500 *μ*M), suggesting that CUR regulated the dysfunction of osteoblastic MC3T3-E1 cells caused by excess iron.

### 3.3. CUR Increases the Antioxidant Capacity in Iron-Overload Mice and Osteoblastic MC3T3-E1 Cells Treated with Excess Iron

**Figure 3:**
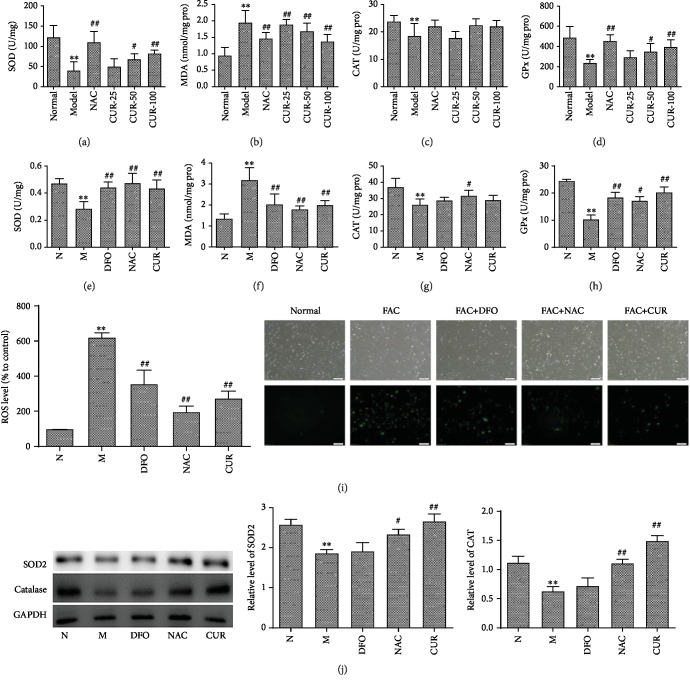
The antioxidant effect of CUR in iron-overload mice and MC3T3-E1 cells treated with excess iron. (a–d) The values of SOD, MDA, CAT, and GPx in the liver tissue of iron-overload mice assayed by ELISA. (e–h) The levels of SOD, MDA, CAT, and GPx in MC3T3-E1 cells. (i) The inhibitory effect of CUR, NAC, and DFO on the generation of ROS in MC3T3-E1 cells treated with excess iron, upper row: cell vitality; down row: fluorescent intensities of ROS. (j) The protein expression of SOD and CAT in MC3T3-E1 cells. Results are represented as the mean ± SD. (*n* = 10 in animal experiments; *n* = 6 in osteoblast experiments), ^∗^*P* < 0.05 and ^∗∗^*P* < 0.01, compared with the normal control group; ^#^*P* < 0.05 and ^##^*P* < 0.01, compared to the model group. N: normal control group, cells were treated with DMSO; M: model group, cells were treated with FAC (500 *μ*M); DFO: cells were treated with DFO (100 *μ*M) and FAC (500 *μ*M); NAC: cells were treated with NAC (1000 *μ*M) and FAC (500 *μ*M); CUR: cells were treated with CUR (10 *μ*M) and FAC (500 *μ*M).

To further confirm the hypothesis that the bone protective effect of the CUR was primarily caused by antagonizing the iron-overload-induced oxidative damage, the levels of MDA, CAT, SOD, and GPx in the liver of mice were determined. As shown in Figures 3(a)–3(d), excess iron significantly decreased the activities of the antioxidative enzymes CAT, SOD, and GPx, and elevated the level of MDA in the liver of mice. Administration of CUR and antioxidant NAC restored SOD and GPx activities and decreased the level of MDA in the liver tissue of iron-overload mice.

Furthermore, effects of excess iron on oxidative stress of osteoblastic MC3T3-E1 cells and reverse effects of CUR were also investigated by biochemical method and the fluorescence microscope qualitative and fluorescence quantitative analysis. After a 3-day treatment with FAC, the activities of antioxidative enzymes SOD, CAT, and GPx were significantly decreased, and the levels of lipid oxidation product MDA were significantly increased in osteoblastic MC3T3-E1 cells, while the CUR, antioxidant NAC, and DFO significantly enhanced the activities and protein expression of SOD, CAT, and GPx, and decreased the level of MDA in MC3T3-E1 cells treated with excess iron (Figures 3(f)–3(h)). Moreover, treatment with FAC for 24 h significantly enhanced the level of ROS in MC3T3-E1 cells, while treatment with CUR, antioxidant NAC, or DFO can significantly decrease the level of ROS in MC3T3-E1 cells (Figure 3(i), Supplement [Supplementary-material supplementary-material-1]). Western blot results (Figure 3(j)) also found treatment with CUR, NAC, or DFO can improve the protein expression of SOD and CAT, which were inhibited by excess iron in MC3T3-E1 cells. The above results indicated that CUR enhanced antioxidative capacities and reduced oxidative damage in MC3T3-E1 cells damaged with excess iron.

### 3.4. CUR Increases the Antioxidant Capacity through Regulation of FoxO1, p53, and Nrf-2 Transcriptional Activity in MC3T3-E1 Cells

**Figure 4:**
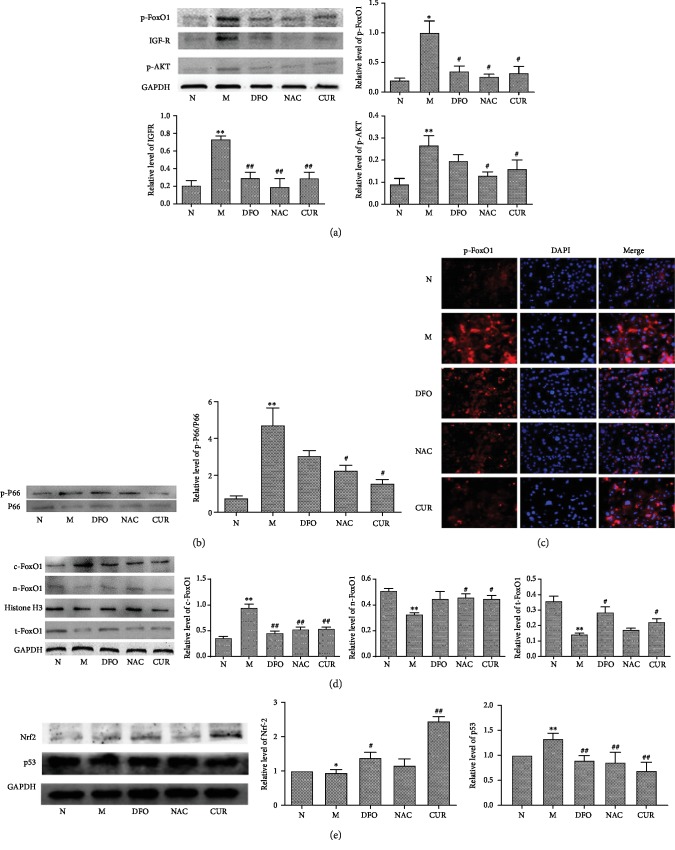
CUR regulates the protein expression in the FoxO1 pathway of osteoblastic MC3T3-E1 cells treated with excess iron. Osteoblastic MC3T3-E1 cells were treated with CUR, NAC, or FAC for 48 h, and after 2 h treatment, cotreated with FAC. Western blot analysis and immunofluorescence staining were used to detect the expression of related proteins in the FoxO1 pathway. (a) Protein levels of p-FoxO1, IGFR, and p-Akt. (b) Protein level of p-p66. (c) Immunofluorescence staining of p-FoxO1. (d) Distribution analysis of FoxO1 in the cytoplasm and nucleus. (e) Protein levels of Nrf2 and p53. Results are represented as the mean ± SD (*n* = 3). ^∗^*P* < 0.05, ^∗∗^*P* < 0.01 compared with the normal control group; ^#^*P* < 0.05, ^##^*P* < 0.01 compared with the model group. N: normal control group, cells were treated with DMSO; M: model group, cells were treated with FAC (500 *μ*M); DFO: cells were treated with DFO (100 *μ*M) and FAC (500 *μ*M); NAC: cells were treated with NAC (1000 *μ*M) and FAC (500 *μ*M); CUR: cells were treated with CUR (10 *μ*M) and FAC (500 *μ*M).

The molecular mechanism underlying antioxidation in iron-overload MC3T3-E1 cells by CUR treatment was further investigated. FoxO1 and its upstream regulatory genes p66, JNK, GLUT1, GLUT3, IGFR, and Akt were analyzed using Western blotting. After a 3-day treatment with 500 *μ*M FAC, the expression of p66, JNK, IGFR, and Akt was elevated, and the expression of GLUT1, GLUT3, and FoxO1 was reduced in MC3T3-E1 (Supplement [Supplementary-material supplementary-material-1]). As shown in Figures 4(a) and 4(b), CUR decreased the expression of IGFR and phosphorylation of Akt, p66, and FoxO1, decreased the cytoplasmic expression of FoxO1, and increased the nuclear expression of FoxO1 in iron-overload MC3T3-E1 cells (Figure 4(d)). These findings were also consistent with the result of immunohistochemical analysis of p-FoxO1 in iron-overload MC3T3-E1 cells (Figure 4(c)) that when FoxO1 was phosphorylated, it was excluded from the nucleus.

P53 play a critical role in apoptosis induced by oxidative stress in MC3T3-E1 cells [20]. NF-E2-related factor 2 (Nrf2) signaling also is involved in the potent antioxidant signaling [21]. Western blot analysis (Figure 4(e)) demonstrated that excess iron significantly inhibited the expression of Nrf2 and increased expression of p53 in MC3T3-E1 osteoblastic cells, while treatment with CUR, DFO, or NAC significantly decreased the expression of p53 and enhanced the expression of Nrf2 in MC3T3-E1 cells treated with excess iron. The above results demonstrated that CUR modulated oxidative stress of osteoblastic MC3T3-E1 cells induced by excess iron via the AKT downstream pathway FoxO1, Nrf2, and p53.

### 3.5. CUR Increases the Expression of FoxO1 Target Genes, Enhances Cell Viability and Autophagy, and Inhibits Apoptosis in MC3T3-E1 Cells Treated with Excess Iron

**Figure 5:**
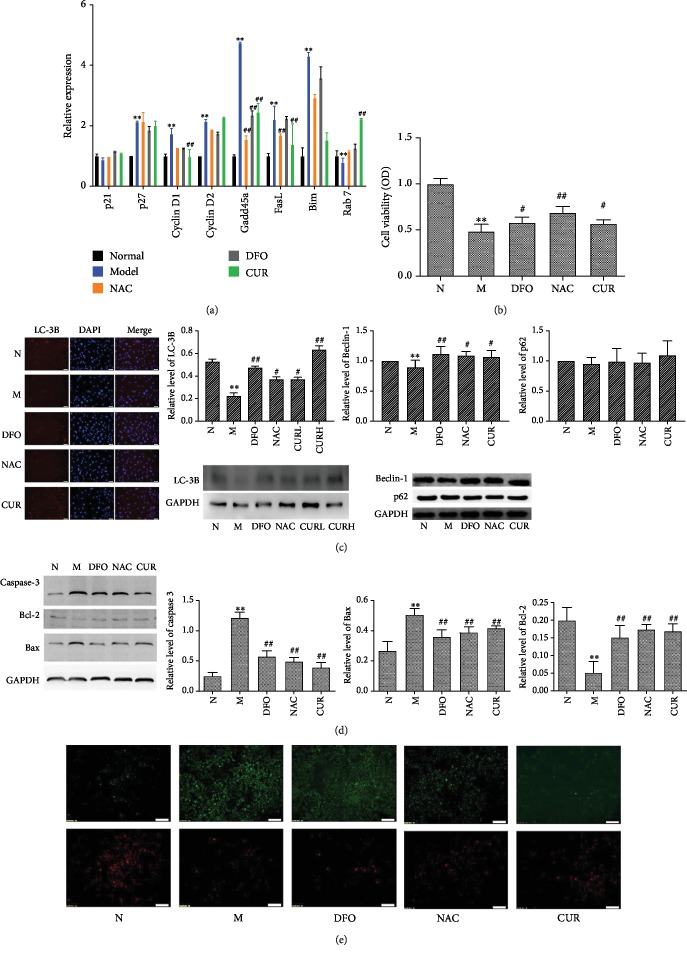
Effects of CUR on the expression of FoxO1 target gene as well as cell viability, apoptosis, and autophagy in MC3T3-E1 cells treated with excess iron. Osteoblastic MC3T3-E1 cells were treated with CUR, NAC, or FAC for 48 h, and after 2 h treatment, cotreated with FAC. (a) The expression of FoxO1 target genes p21, p27, cyclin D1, cyclin D2, Gadd45a, FasL, Bim, and Rab7 was detected with RT-qPCR analysis. (b) Cell viability was determined by MTT assay, and the morphological changes of cell confluence were observed under a phase-contrast microscope (×100). (c) The expression of autophagy proteins LC-3B, Beclin-1, and p62 was detected by Western blot and immunohistochemical staining (LC-3B). (d) Apoptosis-related proteins including caspase 3, Bcl-2, and Bax were detected by Western blot. (e) The mitochondrial membrane potential was detected with JC-1 assay kits. Results are represented as the mean ± SD (*n* = 3). ^∗^*P* < 0.05, ^∗∗^*P* < 0.01 compared with the normal control group; ^#^*P* < 0.05, ^##^*P* < 0.01 compared with the model group. N: normal control group, cells were treated with DMSO; M: model group, cells were treated with FAC (500 *μ*M); DFO: cells were treated with DFO (100 *μ*M) and FAC (500 *μ*M); NAC: cells were treated with NAC (1000 *μ*M) and FAC (500 *μ*M); CUR: cells were treated with CUR (10 *μ*M) and FAC (500 *μ*M).

Firstly, FoxO1 translocates into the nucleus to control the expression of its target genes, such as cyclin D1, cyclin D2, p27, Gadd45a, Bim, Fasl, and Rab7 to regulate the proliferation, differentiation, and apoptosis of cells. Knowing that CUR could increase the expression of FoxO1 in the nucleus of MC3T3-E1 cells, the effect of CUR on target genes of MC3T3-E1 cells injured by excess iron was determined by real-time PCR analysis. The results (Figure 5(a)) showed that excess-iron treatment enhanced the expression of p27, cyclin D1, cyclin D2, Gadd45a, Fasl, and Bim and reduced the expression of Rab7 in MC3T3-E1 cells, and CUR could decrease the expression of cyclin D1, Gadd45a, Fasl, and Bim, and increase the expression of Rab7 in MC3T3-E1 cells damaged with excess iron. The expression and activity pattern of FoxO1-regulated genes indicated that CUR could increase antioxidant defense responses, enhance autophagy, and decrease apoptosis in iron-overload MC3T3-E1 cells.

Knowing that FoxO1 modulates downstream target genes to control cell viability and proliferation, we investigated the effect of CUR on MC3T3-E1 cells treated with excess iron and found that excess-iron treatment decreased the viability of MC3T3-1 cells, while CUR, NAC, and DFO treatment attenuated the inhibitory effect of excess iron on viability of osteoblasts as evidenced by MTT assay of MC3T3-1 cells (Figure 5(b)).

Knowing that autophagy is affected by oxidative stress and plays an important role in osteoblast mineralization and bone homeostasis, the autophagy-associated proteins, such as Beclin-1 (Atg6), p62, and the microtubule-associated protein 1 light chain 3B (LC3B), were determined by Western blot assay and immunofluorescence staining (LC3B). As shown in Figure 5(c), FAC treatment significantly reduced the expression of LC3B and Beclin-1 protein in osteoblastic MC3T3-E1 cells, while CUR, DFO, or NAC reversed the altered expression of LC3B and Beclin-1 protein caused by FAC. The expression of p62 was reduced by FAC and reversed by CUR, DFO, or NAC, but with no significant difference. The above results indicated that CUR counteracted the oxidative injury to the osteoblasts induced by excess iron by enhancing autophagy.

Knowing that FoxOs could inhibit osteoblast apoptosis by suppressing oxidative stress, we used Western blot and mitochondrial membrane potential assay to evaluate the effect of CUR on apoptosis of osteoblasts damaged by excess iron. As shown in Figures 5(d) and 5(e), FAC treatment upregulated the proapoptotic protein Bax, downregulated antiapoptotic protein Bcl-2, activated their downstream protein pro-caspase-3, and reduced the mitochondrial membrane potential in osteoblastic MC3TC cells. CUR, NAC, or DFO treatment could reverse the alteration of Bax, Bcl-2, caspase-3, and mitochondrial membrane potential in MC3T3 cells, indicating that CUR could inhibit apoptosis of osteoblasts in excess-iron-induced oxidative stress.

## 4. Discussion

CUR is one of the main bioactive phenolic compounds in *Curculigo orchioides* Gaertn. It is reported to possess the antioxidant, anti-inflammatory, and free radical scavenging properties. CUR has also been demonstrated to have a bone-protective effect probably due to its antioxidative activity [10]. However, details about the molecular mechanism underlying these protective effects of CUR are not clearly understood. It was found in the present study that CUR could prevent bone loss in iron-overload mice, mitigate injuries to osteoblastic MC3T3-E1 stimulated by oxidative stress, and reduce the production of excess ROS resulting from excess iron. These protective effects of CUR on bone loss and reduced osteoblastic bone formation are believed to be related to the AKT-FoxO1 pathway.

Oxidative stress plays an important role in bone metabolisms by generating excess ROS to inhibit osteoblastic bone formation and increasing osteoclastic bone resorption, eventually leading to bone loss and osteoporosis [18]. Excess iron can damage cellular components by catalyzing the formation of high ROS such as hydroxyl radicals. Accumulating evidence indicates that iron overload has a direct detrimental effect on bone metabolism. A 3-year retrospective longitudinal study [16] found that iron-overload accelerated bone loss in healthy postmenopausal women and middle-aged men. Iron overload increased oxidative stress and bone resorption, and altered the bone microarchitecture and mechanical properties in a mouse model [22]. In the present study, we successfully established a bone loss mouse model through 3-month administration of iron dextran (1.0 g/kg) and found that iron overload induced iron accumulation in the tissue, increased the ROS level, and decreased the activities of antioxidative enzymes in mice. In addition, iron overload also caused disturbance of bone metabolism as evidenced by serum biochemical parameters (reduced bone formation and increased bone resorption) and deterioration of the mouse femoral bone trabecular architecture. Treatment of iron-overload mice with the antioxidant NAC or CUR could both decrease the oxidative stress and bone loss, and attenuate the development of trabecular and cortical bone abnormalities, demonstrating that CUR could alleviate the bone loss in mice induced by oxidative stress resulting from iron overload. Compared with the positive control group (NAC), CUR had the same or even better antiosteoporosis effect, but with much lower dose.

Oxidative stress reduces the osteogenic effect of osteoblasts. In vitro studies [23] demonstrated that excess iron inhibited the proliferation and differentiation of osteoblasts by enhancing mitochondrial biogenesis. Our study found that treatment with excess iron increased the ROS level, inhibited the proliferation and expression of osteogenic phenotyoic markers such as Runx2, ALP, and OCN, and decreased mineralization of the bone matrix. The iron chelator DFO and antioxidant NAC reversed the alteration of osteoblasts treated with excess iron. CUR could inhibit the production of ROS, increase the activities of antioxidant enzymes SOD and CAT, and reverse the reduction of Runx2, OCN, and type 1 collagen in osteoblasts damaged by excess iron, indicating that CUR could ameliorate oxidative injures in osteoblastic MC3T3-E1 cells induced by excess iron.

FoxO1 plays an important role in regulating redox balance and the function of osteoblasts. Oxidative stress induces generation of H_2_O_2_ and initiates signal cascade reaction to regulate ERKs, JNKs, p38 MAPK, and the PI3K/Akt pathway, thus improving the expression of p53 and p66shc and subsequently decreasing the proliferation, differentiation, and mineralization of osteoblasts [24, 25]. FoxO1 can translocate from the cytoplasm into the nucleus to increase the activities of antioxidative enzymes SOD and CAT to mitigate the oxidative stress. P66shc, which is the key protein to stimulate oxidative stress in the mitochondrial membrane, induced cell apoptosis by increasing the generation of H_2_O_2_ and disturbing redox equilibrium [26]. The present study finds that iron overload stimulates oxidative stress and damages osteoblasts through modulating the ROS/p66shc/JNK pathway. FAC treatment MC3T3-E1 cells for 6-72 h increase the phosphorylation of both p66 and JNK and generation of ROS. JNK regulates the phosphorylation and distribution of FoxO1 in the cytoplasm and nucleus. The phosphorylated JNK inhibits the phosphorylation of FoxO1 and promotes FoxO1 translocation into the nucleus, and then increases the activities of antioxidative enzymes SOD and CAT to mitigate the oxidative stress. In addition, persistent oxidative stress decreases the protein levels of GLUT1 and GLUT3 (the key protein of glucose metabolism) to cause dysfunction of energy metabolism in osteoblast and compensatory overactivation of IGFR and activate the phosphorylation of AKT, finally leading to the degradation of FoxO1. Therefore, oxidative stress resulting from persistent iron overload caused a relative increase of FoxO1 in the cytoplasm and a relative reduction in the nucleus of osteoblast, which may be related with increased phosphorylation of FoxO1 and its degradation in the nucleus. The iron chelator DFO and antioxidant NAC reversed the alteration of distribution of FoxO1 and decrease the oxidative injury osteoblasts. CUR attenuated the effect of iron overload on FoxO1, enhanced the protein level of FoxO1, and regulated the distribution of FoxO1 in the nucleus, thus improving the antioxidative capacities in MC3T3-E1-cells.

In addition, excess iron increased the expression level of FoxO1 in the early phase, and CUR decreased the expression level of FoxO1 in MC3T3-E1 cells after 72 h treatment. Persistent iron overload resulted in sustained oxidative stress and subsequently caused the overactivation of IGFR and AKT and hyperphosphorylation of FoxO1, finally impairing the function of osteoblasts. CUR inhibited the overactivation of IGFR and AKT and the phosphorylation of FoxO1 and p66, improved the translocation of FoxO1 into the nucleus, and increased the expression of target genes SOD and CAT to counteract the oxidative injury to osteoblasts. Further studies showed that CUR also can activate Nrf2 signaling, downstream of AKT, to inhibit excess-iron-induced reactive oxygen species (ROS) production in MC3T3-E1 cells.

During the process of translocating from the cytoplasm into the nucleus, FoxO1 also modulates the gene expression, such as Gadd45 for DNA repair, cyclins D1 and D2 for cell cycle control, p27kip1 for cell cycle arrest, Bim and FasL for apoptosis, and Rab7 for autophagy, subsequently to antagonize oxidative stress and functional damage to osteoblasts. In this study, we found that a long-term (24-72 h) iron-overload condition increased the expression of cyclin D1, Gadd45a, Bim, and FasL and decreased the expression of SOD and Rab7, while CUR decreased the expression of cyclin D1, Gadd45a, Bim, FasL, and elevated the expression of SOD and Rab7 in MC3T3-E1 cells treated with excess iron, indicating that CUR may be involved in the viability, autophagy, and apoptosis of osteoblasts damaged by excess iron.

Autophagy is a lysosomal degradation and self-digesting process that protects cells against various cytotoxic stimuli by degrading damaged organelles. Autophagy is believed to protect against cell death under the condition of oxidative stress. FoxO1 and p53 are also involved in the regulation of autophagic gene. High concentrations of iron can increase the expression of Beclin-1 and LC3 II while reducing the expression of p62 for short-term exposure (24 h-48 h) [27], but in this experiment, long treatment (3-5 days) with excess iron can decrease the expression of autophagic protein LC-3B and Beclin-1 in osteoblastic MC3T3-E1 cells, indicating that iron overload for long-term exposure can induce the generation of ROS and decrease the autophagy of osteoblast. While treatment with CUR, NAC, or DFO, the expression of LC-3B and Beclin-1 was increased, indicating that CUR can elevate the capacity of osteoblastic bone formation under oxidative stress through increasing autophagy via the FoxO1 pathway [28].

FoxO1 regulates the expression of apoptosis-related gene in osteoblast [23]. Previous investigations demonstrated that FoxO1 mRNA expression in Bcl2-/-calvariae osteoblasts was upregulated and the phosphorylation of THr24 in FoxO1 was reduced [29], and that the expression of FoxO1 target genes including FasL, Gadd45a, and Bim was upregulated [30]. The present study found that iron overload upregulated the expression of apoptotic gene caspase-3, Bax, and FoxO1 target genes Fasl and Bim in osteoblastic MC3T3-E1 cells, and inhibited the expression of antiapoptotic gene Bcl-2 in osteoblastic MC3T3-E1 cells. CUR reversed the altered expression of apoptosis-related genes, indicating that CUR inhibited the apoptosis in osteoblast treated with excess iron via the FoxO1 pathway.

Some clinical studies [22, 31] revealed that osteoporosis and bone fracture occurred frequently in patients with iron-overload-related diseases such as hemoglobinopathies, thalassemia, hereditary hemochromatosis, and sickle cell disease. The iron concentration in the human body also increases rapidly in menopausal women and male adolescents. Although these age-related iron accumulation appears to be moderate compared with iron-overload-associated diseases, mounting evidence indicates that other than estrogen deficiency, iron overload is an independent risk factor for osteoporosis [32]. In addition, a number of experimental animal models of iron overload have been established to confirm the deleterious effect of iron overload on bone metabolism. It was found that iron-overload-driven bone loss was associated with the increased production of ROS. The free radical scavenger NAC could partially prevent the development of iron-overload-triggered bone abnormalities, highlighting the critical role of ROS in the mechanism of bone loss [13]. Moreover, iron chelator desferrioxamine (DFO) attenuated the antiosteogenic effect of iron overload, demonstrating that free iron plays a crucial role in inhibiting osteogenic differentiation of MSCs [33]. Hence, the bone-targeted iron chelator exhibits therapeutic potential to protect against bone loss associated with age-iron accumulation as well as iron-overload-related diseases. The present investigation found that CUR, antioxidant NAC, and DFO could counteract the damage of iron overload on bone metabolism and decrease bone loss. The antioxidant NAC activated the differentiation and mineralization of osteoblast treated with excess iron either directly or by counteracting the action of the oxidant. DFO diminished the excess iron to reduce the oxidative injury. CUR, a polyphenol compound, possesses antioxidative and osteogenic activities and was able to attenuate bone loss in iron-overload mice at a relatively lower dose through mediation of the FoxO1 signal pathway.

In summary, CUR protects against excess-iron-induced bone loss primarily by ameliorating the loss of FoxO1, decreasing phosphorylation of FoxO1, and improving the translocation to nuclear through inhibiting the IGFR/Akt pathway. Given the outstanding antioxidant and antiapoptotic and autophagy-promoting properties of CUR, it may prove to be a promising candidate for preventing and delaying the progression of osteoporosis.

## Figures and Tables

**Table 1:** Effects of CUR on femoral BMD, architecture, and mechanical properties in iron-overload mice.

	Control	Iron treatment	NAC (500 mg/kg)	CUR (25 mg/kg)	CUR (50 mg/kg)	CUR (100 mg/kg)
Micro-CT						
BMD (Au/mm^3^)	302.3 ± 52.6	185.6 ± 17.4^∗^	256.5 ± 27.0^##^	205.2 ± 27.3	210.5 ± 26.4	286.3 ± 11.3^##^
BS/BV (%)	46.07 ± 7.61	62.47 ± 5.94^∗^	46.26 ± 2.43^##^	60.74 ± 4.35	55.64 ± 2.00	51.02 ± 5.70^#^
BVF (%)	25 ± 7	12 ± 2^∗^	21 ± 3^##^	12 ± 2	15 ± 0.02	21 ± 6^#^
Trabecular bone						
Trabecular number (N/mm)	5.58 ± 1.26	3.54 ± 0.34^∗^	4.83 ± 0.77^##^	3.69 ± 0.44	4.21 ± 0.56	5.27 ± 1.08^#^
Trabecular spacing (*μ*m)	0.14 ± 0.04	0.25 ± 0.03	0.17 ± 0.03^##^	0.24 ± 0.03	0.20 ± 0.03	0.16 ± 0.05^#^
Trabecular thickness (*μ*m)	0.044 ± 0.008	0.032 ± 0.003^∗^	0.043 ± 0.002^##^	0.033 ± 0.002	0.036 ± 0.001	0.040 ± 0.005^#^
Cortical bone						
Cortical thickness (mm)	0.135 ± 0.005	0.117 ± 0.004^∗^	0.134 ± 0.003^##^	0.125 ± 0.004	0.123 ± 0.003	0.126 ± 0.004^##^
Cortical area (mm^2^)	0.80 ± 0.03	0.61 ± 0.01^∗^	0.73 ± 0.01^##^	0.70 ± 0.06	0.71 ± 0.05	0.79 ± 0.05^#^
Marrow area (mm^2^)	1.20 ± 0.01	1.33 ± 0.03^∗^	1.27 ± 0.01^##^	1.30 ± 0.04	1.29 ± 0.04	1.25 ± 0.02^##^
Mechanical properties						
Elastic load (N)	14.04 ± 1.67	9.25 ± 0.80^∗^	10.49 ± 1.80	8.52 ± 0.31	10.22 ± 1.72	11.80 ± 0.94^##^
Elastic stress (N/mm^2^)	33.90 ± 3.35	24.28 ± 4.11^∗^	32.21 ± 4.44^#^	22.87 ± 2.17	25.64 ± 4.10	29.50 ± 1.85
Elastic modulus (Gpa)	1.49 ± 0.35	0.84 ± 0.20^∗^	1.33 ± 0.30^#^	0.91 ± 0.28	1.03 ± 0.21	1.23 ± 0.31^#^
Maximum load (N)	16.57 ± 2.71	11.65 ± 2.35∗	13.25 ± 1.31	9.52 ± 0.87	12.47 ± 2.07	13.46 ± 1.07
Maximum stress (N/mm^2^)	41.62 ± 2.25	26.65 ± 4.94^∗^	39.59 ± 8.04^#^	25.55 ± 3.38	31.48 ± 6.54	34.35 ± 2.13^#^

Results are represented as the mean ± SD (*n* = 10). ^∗^*P* < 0.01, compared to the control group; ^#^*P* < 0.05, ^##^*P* < 0.01, compared to the iron-treated group.

## Data Availability

The (data type) data used to support the findings of this study will be included within the supplementary information file(s).

## Abbreviations

CUR: Curculigoside
MnSOD: Manganese superoxide dismutase
FoxO1: Forkhead box protein O1
ROS: Reactive oxygen species
NAC: *N*-acetyl-L-cysteine
Nrf2: NF-E2-related factor 2
DFO: Desferrioxamine
FAC: Ferric ammonium citrate
ColI: Type I collagen
Runx2: Runt-related transcription factor 2
OCN: Osteocalcin
OPG: Osteoprotegerin
RANKL: Receptor activator for nuclear factor-*κ* B ligand
CAT: Catalase
GPx: Glutathione peroxidase
MDA: Malondialdehyde
JNK: c-Jun N-terminal kinase
GLUT1: Glucose transporter 1
GLUT3: Glucose transporter 3
IGFR: Insulin-like growth factor receptor 1
Akt: Protein kinase B
Bcl-2: B cell lymphoma-2
BAX: Apoptosis regulator BAX
LC3B: Microtubule-associated protein 1 light chain 3 *β*
GAPDH: Glyceraldehyde-3-phosphate dehydrogenase.

## Supplementary Materials

Supplementary Materials Supplementary Table S1: primers used for sequences for RT-qPCR. Supplement Figure 1: cell fluorescence intensity detected by a fluorescence microscope. The level of reactive oxygen species in MC3T3-E1 cell was significantly increased from 1 to 24 h after intervention by FAC (500 *μ*M). Supplement Figure 2: the effect of oxidative stress induced by iron overload on the upstream of FoxO1 in osteoblasts. (A) The change of p66 phosphorylation level in osteoblasts induced by iron overload with time. (B) The change of JNK phosphorylation level in osteoblasts induced by iron overload with time. (C) The change of GLUT1 level in osteoblasts induced by iron overload with time. (D) The change of GLUT3 level in osteoblasts induced by iron overload with time. (E) The change of IGFR level in osteoblasts induced by iron overload with time. (F) The change of AKT phosphorylation level in osteoblasts induced by iron overload with time. Data represent the mean ± SD, *n* = 3, ^∗^*P* < 0.05, ^∗∗^*P* < 0.01 vs. normal. ^#^*P* < 0.05, ^##^*P* < 0.01 vs. model.
