# Supplementary material for: Curculigoside Protects against Excess-Iron-Induced Bone Loss by Attenuating Akt-FoxO1-Dependent Oxidative Damage to Mice and Osteoblastic MC3T3-E1 Cells
Source: Oxid Med Cell Longev. 2019 Dec 17;2019:9281481. doi: 10.1155/2019/9281481 (PMC6948300; doi:10.1155/2019/9281481)
Supplement: Supplementary Materials — Supplementary Table S1: primers used for sequences for RT-qPCR. Supplement Figure 1: cell fluorescence intensity detected by a fluorescence microscope. The level of reactive oxygen species in MC3T3-E1 cell was significantly increased from 1 to 24 h after intervention by FAC (500 μM). Supplement Figure 2: the effect of oxidative stress induced by iron overload on the upstream of FoxO1 in osteoblasts. (A) The change of p66 phosphorylation level in osteoblasts induced by iron overload with time. (B) The change of JNK phosphorylation level in osteoblasts induced by iron overload with time. (C) The change of GLUT1 level in osteoblasts induced by iron overload with time. (D) The change of GLUT3 level in osteoblasts induced by iron overload with time. (E) The change of IGFR level in osteoblasts induced by iron overload with time. (F) The change of AKT phosphorylation level in osteoblasts induced by iron overload with time. Data represent the mean ± SD, n = 3, ∗P < 0.05, ∗∗P < 0.01 vs. normal. #P < 0.05, ##P < 0.01 vs. model. [file 9281481.f1.pdf]

Supplementary table S1. Primers used for sequences for RT-qPCR

Gene	Forward primers(5’-3’)	Reverse primers (5’-3’)
Cyclin D1	CGTATCTTACTTCAAGTGCGTG	ATGGTCTCCTTCATCTTAGAGG
Cyclin D2	GTCTGTGAGGAACAAAAGTGTG	CTGAAGATGGGTCTTAGGAGTC
Gadd45a	ATGACTTTGGAGGAATTCTCGG	GGATGAGGGTGAAATGGATCTG
Bim	ATCCCCGCTTTTCATCTTTA	AGGACTTGGGGTTTGTGTTG
P27	TTTAATTGGGTCTCAGGCAAAC	CCCTTTTGTTTTGCGAAGAAGA
P21	ATGTCCAATCCTGGTGATGTC	GAAGTCAAAGTTCCACCGTTC
Fasl	AAGAAGGACCACAACACAAATCTG	CCCTGTTAAATGGGCCACACT
Sod2	AAGGGAGATGTTACAACTCAGG	GCTCAGGTTTGTCCAGAAAATG
FSP27	GCC CAG TTC CTT CCT TTC TG	AAC ACT CTC TCG CAC ACC TC
RAB7	GGGGA CTCTGGTGTTGGAA	CGCTCCTATTG TGGCTTTGT
GAPDH	B661304	

.

A

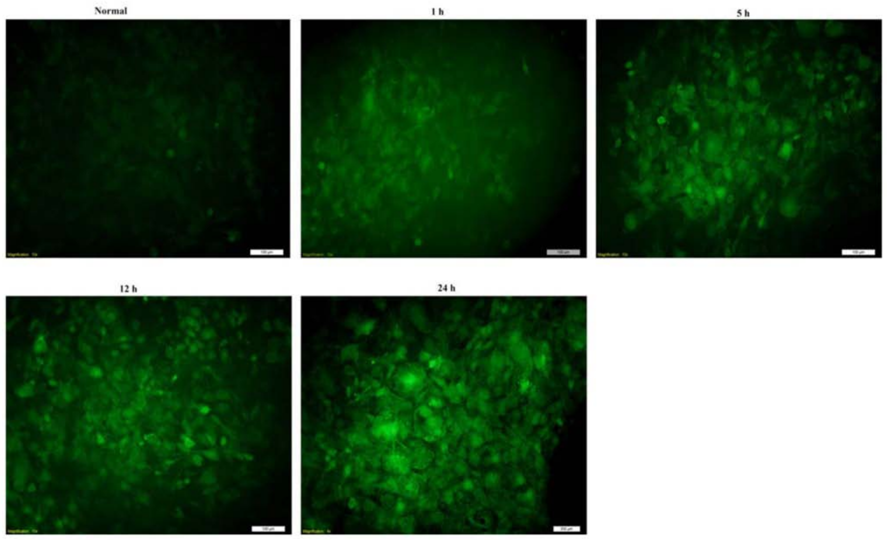

Supplement Figure 1. Cell fluorescence intensity detected by fluorescence microscope. The level of reactive oxygen species in MC3T3-E1 cell was significantly increased from 1 to 24h after intervention by FAC (500μM).

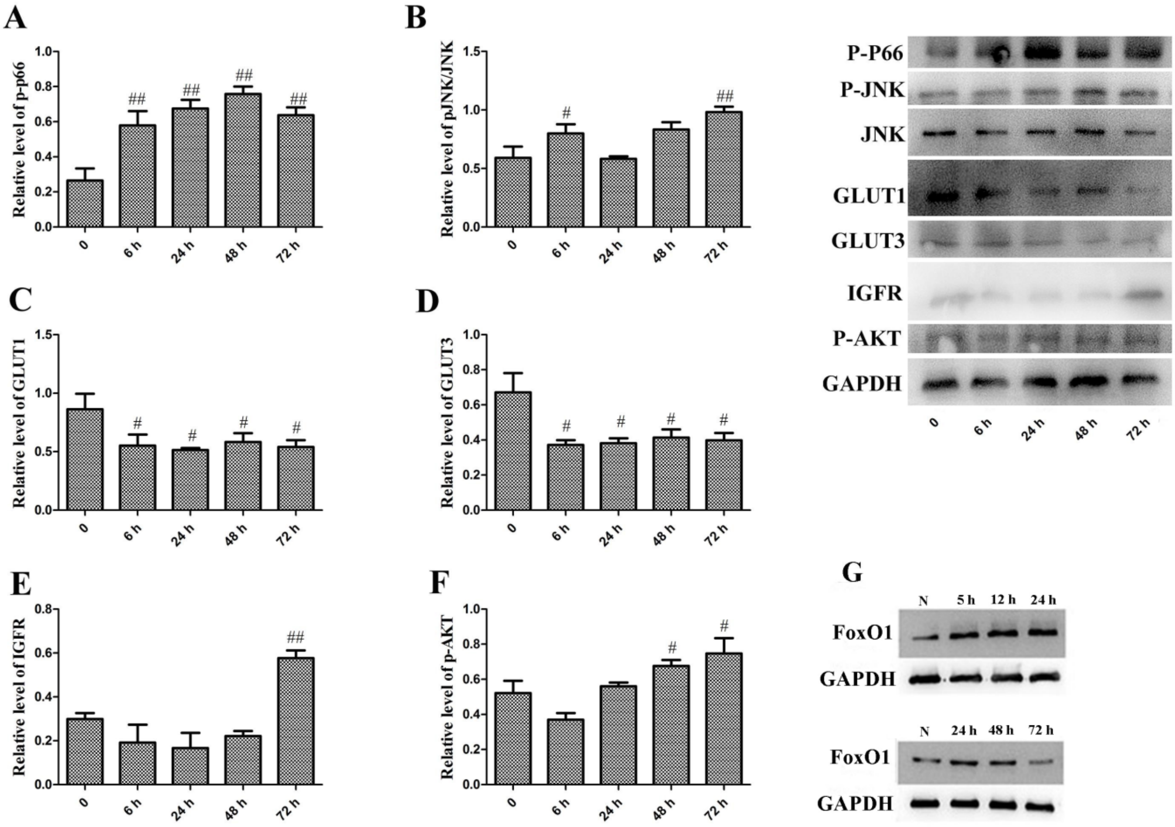

Supplement Figure 2. The effect of oxidative stress induced by iron overload on the upstream of FoxO1 in osteoblasts. (A) The change of p66 phosphorylation level in osteoblasts induced by iron overload with time. (B) The change of JNK phosphorylation level in osteoblasts induced by iron overload with time. (C) The change of GLUT1 level in osteoblasts induced by iron overload with time. (D) The change of GLUT3 level in osteoblasts induced by iron overload with time. (E) The change of IGFR level in osteoblasts induced by iron overload with time.(F) The change of AKT phosphorylation level in osteoblasts induced by iron overload with time. Data represent mean ± SD, n

=3, *P < 0.05, **P < 0.01 vs. normal. #P < 0.05, ##P < 0.01 vs. model.）
